# Effect of kinesiology tape application direction on quadriceps strength

**DOI:** 10.1097/MD.0000000000011038

**Published:** 2018-06-15

**Authors:** Im-Rak Choi, Jung-Hoon Lee

**Affiliations:** a Department of Biomedical Health Science, Graduate School, Dong-Eui University; b Department of Rehabilitation Therapy Team, Sports Exercise Therapy Center, Good Samsun Hospital; c Department of Physical Therapy, College of Nursing and Healthcare Sciences and Human Ecology, Dong-Eui University, Busan, Republic of Korea.

**Keywords:** elastic therapeutic tape, kinesiology tape, peak torque, strength, tape direction

## Abstract

**Background:**

Controversy exists regarding whether the kinesiology tape application direction affects muscle strength.

**Methods:**

Eighteen healthy volunteers (12 men, 6 women) participated. Kinesiology tape was randomly applied to the quadriceps muscles either from origin to insertion or from insertion to origin. A Biodex isokinetic dynamometer was used to measure the peak torque of the quadriceps pre-and post-taping.

**Results:**

There was a significant difference in muscle strength after taping, regardless of the kinesiology tape application direction. There were no significant differences in the peak torque of the quadriceps between the 2 kinesiology tape application directions.

**Conclusions:**

The application of kinesiology tape application to the rectus femoris, vastus medialis, and vastus lateralis of the quadriceps increased the muscle torque, regardless of the tape application direction. Therefore, to enhance quadriceps strength, we recommend the application of kinesiology tape to 3 of the muscles of the quadriceps (specifically, the rectus femoris, vastus medialis and vastus lateralis), irrespective of the tape application direction.

## 1 Introduction

Kinesio taping is a treatment approach using kinesiology tape, and was developed by Kase Kenzo. In the approach, kinesiology tape is applied directly to the skin to treat musculoskeletal injuries.^[1]^ When kinesiology tape is applied to the skin, the tactile stimulus stimulates the afferent nerves,^[2]^ along with stimulating the mechanoreceptors of the skin, joints, muscles, or tendons, to enhance proprioception.^[3,4]^ Kinesiology taping has been reported to be effective in preventing injuries,^[5]^ aiding with rehabilitation^[6]^ and performance improvement,^[7]^ improving pain,^[8,9]^ facilitating joint exercises,^[10–12]^ increasing muscle activation,^[13,14]^ and enhancing muscle strength.^[15,16]^

Kase Kenzo argued that muscles are facilitated when the kinesiology tape application direction is from the origin to the insertion, and are inhibited when the direction is from the insertion to the origin.^[1]^ However, the effect of kinesiology tape application direction remains controversial. Vercelli et al^[17]^ found that quadriceps strength did not differ according to kinesiology tape application direction. In addition, Serrão et al^[18]^ reported that muscle activation during squatting exercises after kinesiology tape treatment was not significantly different between different directions of kinesiology tape application. In contrast, Fukui et al^[19]^ observed that gluteus maximus muscle strength was different according to the kinesiology tape application direction.

Therefore, the present study was conducted to examine whether quadriceps strength differs depending on the kinesiology tape application direction, using isokinetic equipment.

## 2 Method

### 2.1 Subjects

G-Power 3.1 (University of Dusseldorf, Dusseldorf, Germany) was used to determine the sample size required for conducting a 2-tailed paired *t*-test to examine the effect of kinesiology tape application on muscle strength, as well as a 2-tailed independent *t*-test to examine the difference in the effect of kinesiology tape application on muscle strength, according to kinesiology tape application direction.^[20]^ Assuming a significance level of 0.05, a power of 80%, and an effect size of 0.8, the estimated sample size was n = 15. Thus, a total of 18 subjects, aged 20 years or older (12 men and 6 women), who consented to participate in the study were included. Exclusion criteria were limitations in everyday activities due to knee pain, a history of surgery, joint malformations, and back pain. The study was approved by the Institutional Review Board of Dong-Eui University (DIRB-201612-HR-E-035). The clinical trial was registered under the registration number KCT0002659.

### 2.2 Design

Single-blind and cross-over study design was utilized. The participants were randomly assigned to the groups by volunteers who did not participate in the intervention. In the envelopes, the origin to insertion and the insertion to origin direction of the tape application were selected randomly. The subjects were divided into 2 groups and 1 tape application direction was randomly assigned at the first visit; one week later, the other application direction condition was completed.

### 2.3 Procedure

**Figure 1:**
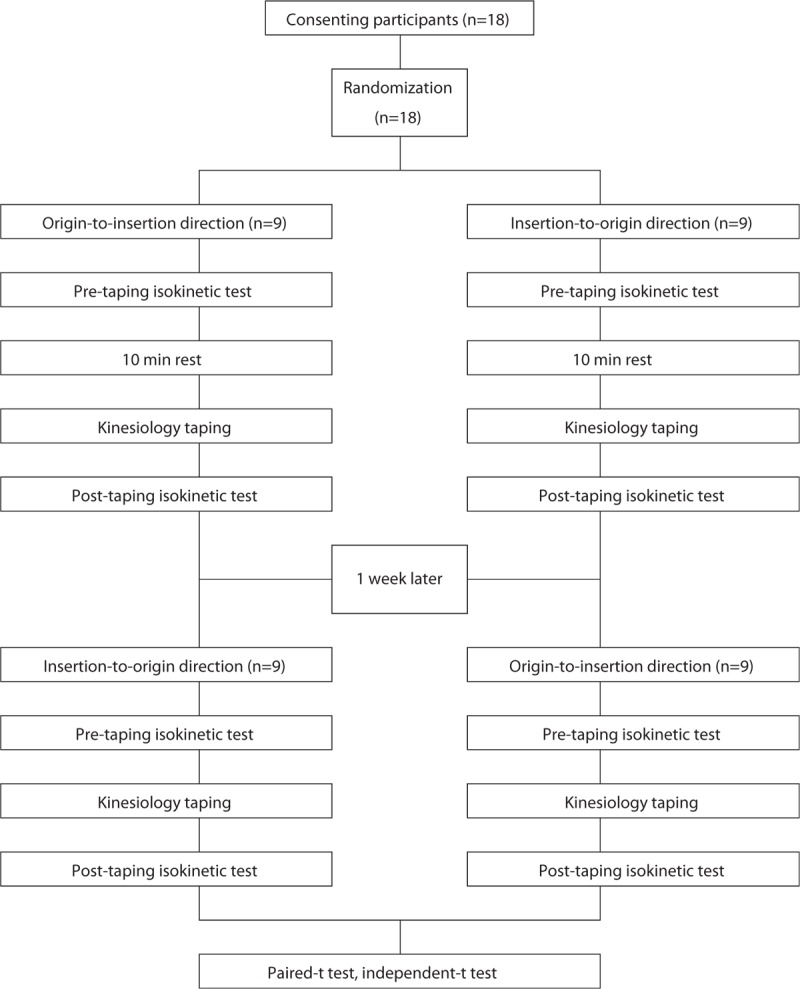
Study flowchart.

The peak torque of the subjects’ quadriceps muscles was measured using isokinetic equipment before the kinesiology tape intervention was performed. After 10 minutes of rest, each group underwent random application of the kinesiology tape application (the origin to insertion or the insertion to origin direction for the applied muscles) and the peak torque was measured once again. One week later, the kinesiology tape was applied in the direction opposite to that of the previous application and the peak torque was measured once again. A flow-chart depicting the experimental procedures and study design is presented in Figure 1.

### 2.4 Isokinetic equipment

Isokinetic equipment (Biodex system 4, Biodex Medical System Inc., New York, NY) was used to investigate the immediate effect of muscle strength increase after kinesiology tape application. Biodex provides constant speed and resistance while a joint is moved within a predetermined range, and draws a curve showing the muscle torque throughout the movement. The highest point on the curve indicates the peak torque. Previous research has demonstrated the high test-retest reliability of this equipment (Intraclass correlation coefficient, 0.82–0.95).^[21,22]^

### 2.5 Kinesiology tape application

With the knee bent at 90°, kinesiology tape (BB Tape, WETAPE Inc., Paju, Korea) was stretched about 20% to 25% of its length, without stretching either end (approximately 2–3 cm), and was applied to 3 of 4 muscles constituting the quadriceps; the rectus femoris, vastus medialis, and vastus lateralis.^[23–26]^

**Figure 2:**
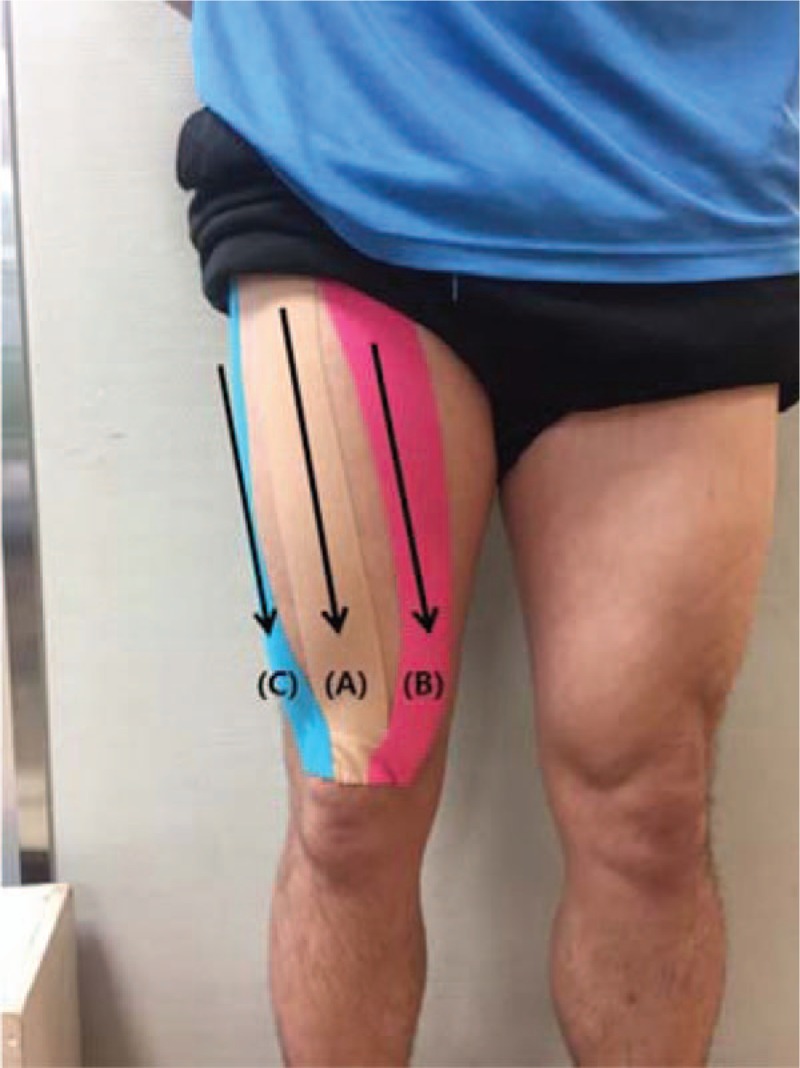
Application of the kinesiology tape in the origin-to-insertion direction.

In the origin to the insertion direction condition, the tapes were applied from the anterior superior iliac spine to the superior border of the patella (the origin and insertion of the rectus femoris, respectively) (Fig. 2A), from inward of the intertrochanteric line to the medial superior aspect of the patella (the origin and insertion of the vastus medialis, respectively) (Fig. 2B), and from the greater trochanter to the lateral superior region of the patella (the origin and insertion of the vastus lateralis, respectively) (Fig. 2C).^[23,27]^

**Figure 3:**
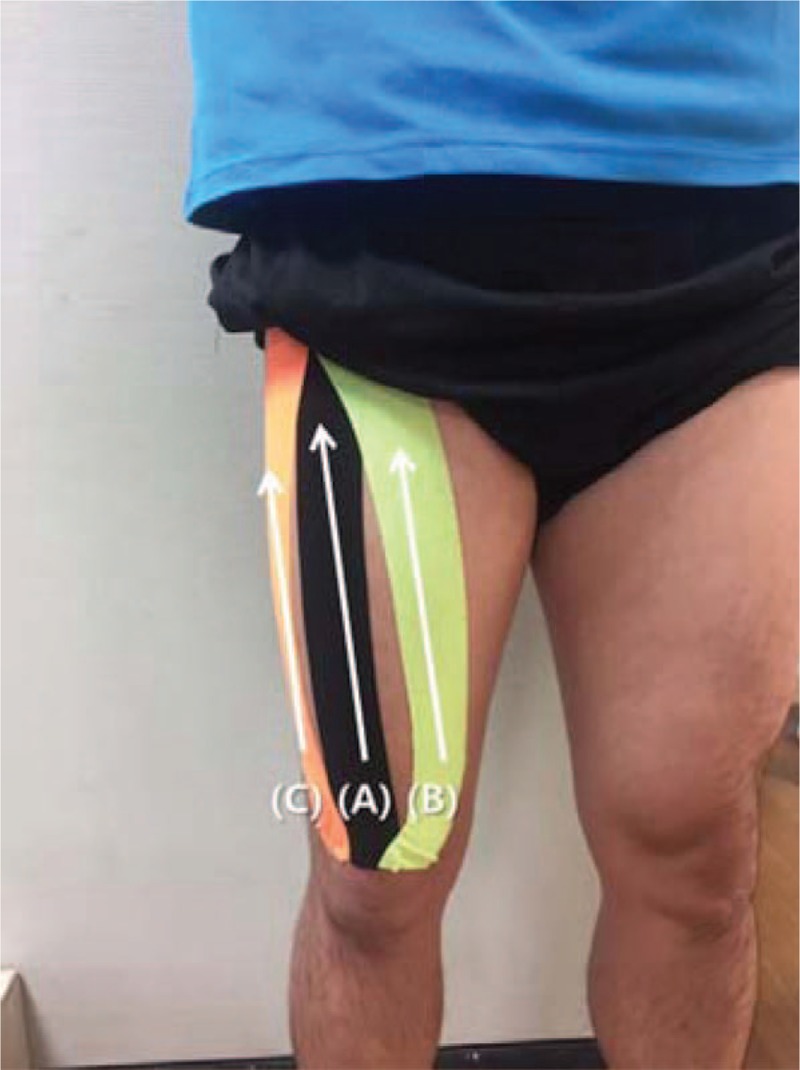
Application of the kinesiology tape in the insertion-to-origin direction.

In the insertion to the origin direction condition, the tapes were applied from the superior border of the patella to the anterior superior iliac spine (the insertion and origin of the rectus femoris, respectively) (Fig. 3A), from the medial superior aspect of the patella to inward of the intertrochanteric line (the insertion and origin of the vastus medialis, respectively) (Fig. 3B), and from the lateral superior region of the patella to the greater trochanter (the insertion and origin of the vastus lateralis, respectively) (Fig. 3C).^[23,27]^

### 2.6 Muscle torque assessment

**Figure 4:**
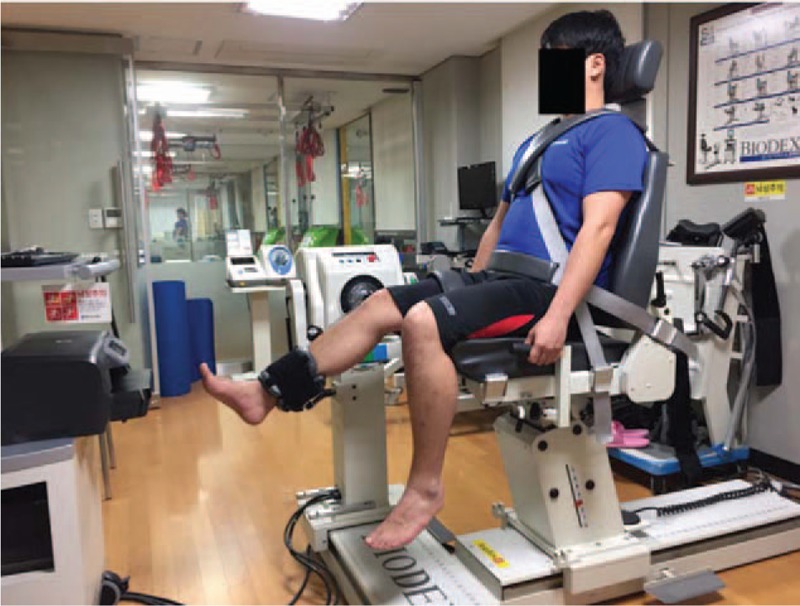
Isokinetic muscle peak torque assessment using the Biodex.

Quadriceps torque was measured using the Biodex before and after kinesiology taping. Subjects first performed low-intensity warm-up exercises, such as stretching and cycling, for 5 minutes.^[15]^ Subjects then sat in the Biodex chair, with their back leaning backwards. The trunk, thighs, and ankles were immobilized via belts, and the knee was fixed to the torque meter (Fig. 4). Biofeedback was provided on a monitor and the experimenter verbally encouraged subjects to use their muscle strength to the maximum. The muscle torque was measured at angular velocities of 60°/s, 120°/s, 180°/s for 10 repetitions each, and the maximal peak torque value was computed across repeated measurements. Furthermore, measurements were taken by applying the specified joint range. Subjects rested for 60 seconds after the measurements for a given angular velocity were completed.^[15]^ After the pre-taping isokinetic test, subjects rested 10 minutes. As inorganic phosphate (Pi) and force in a muscle completely recovers in 5 minutes after holding a contraction at maximum force,^[28]^ this rest period was considered more than adequate. Kinesiology taping was then applied and the subjects completed the post-taping isokinetic test, in the same manner as the pre-taping isokinetic test.

### 2.7 Data analysis

When the Shapiro–Wilk test was performed to confirm the normal distribution, the significance level was greater than .05, and a normal distribution was confirmed. A paired *t*-test was performed to evaluate the effect of kinesiology tape application on muscle peak torque values. In addition, an independent *t*-test was performed to evaluate differences in the effect of kinesiology tape application on muscle peak torque values, according to kinesiology tape application direction. Data were analyzed using SPSS (Version 18.0 for Windows, Chicago, IL). The statistical significance level was set at .05.

## 3 Result

### 3.1 Subject characteristics

Eighteen athletes (12 male and 6 female) volunteered to participate in this study (mean age, 25.89 ± 3.13 years; mean height 168.78 ± 8.7 cm; mean weight 65.39 ± 13.14 kg). All participants were Asian, 61.1% were living with their parents, 33.3% were single, and 5.6% were married. The number of siblings in household was one in 83.3% and zero in the remaining 16.7%. Level of education was 4-year degree in 77.7%, college in 16.7%, and high school in 5.6%. The household income in US dollars was ≤14.999 in 11.1% and 15,000 to 49.000 in 88.9%. The sociodemographic characteristics of subjects are provided in Table 1.

**Table 1:**
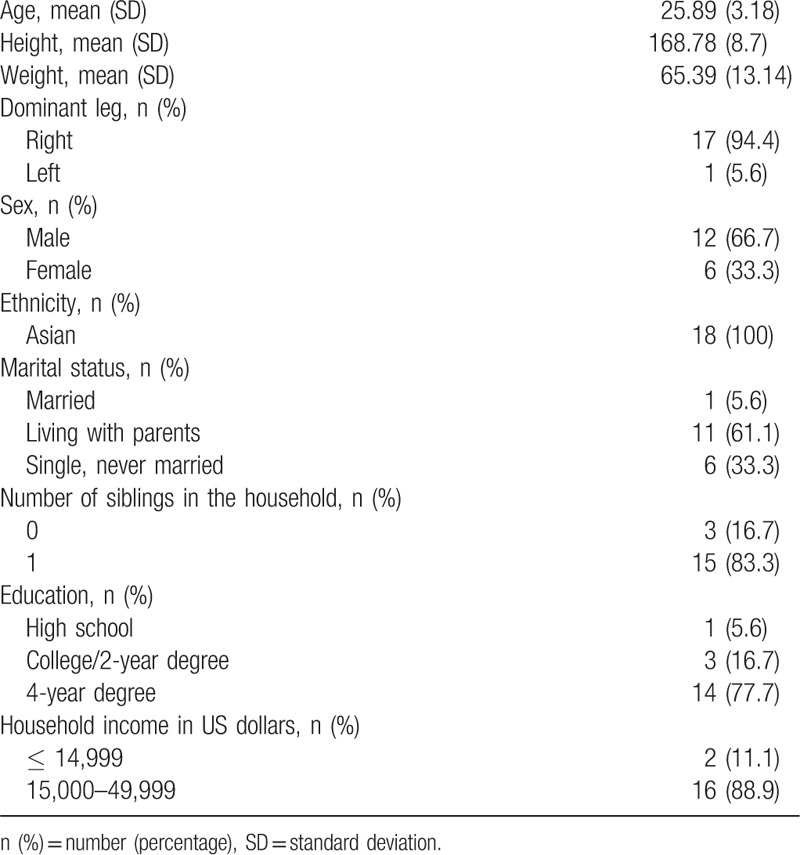
Sociodemographic characteristics of subjects (n = 18).

### 3.2 Change in the peak torque before and after kinesiology tape application

In both tape application direction conditions (from the origin to the insertion and from the insertion to the origin), the peak torque was significantly different after kinesiology tape application compared to that before application (Table 2). At 60°/s, the origin to insertion direction increased from 132.89 ± 59.58 to 151.75 ± 61.59 (*P* = .001), and the insertion to origin direction increased from 138.32 ± 55.94 to 149.14 ± 66.9 (*P* = .016). At 120°/s, the origin to insertion direction increased from 114.7 ± 53.53 to 126.62 ± 51.65 (*P* = .007), and the insertion to origin direction increased from 113.22 ± 49.3 to 119.06 ± 49.53 (*P* = .004). At 180°/s, the origin to insertion direction changed from 92.63 ± 40.34 to 103.05 ± 41 (*P* = .006), and the insertion to origin direction was 91.91 ± 42.07 (*P* = .025). A significant difference was found before and after taping at the angular velocities of 60°/s, 120°/s, and 180°/s, regardless of the tape application direction.

**Table 2:**
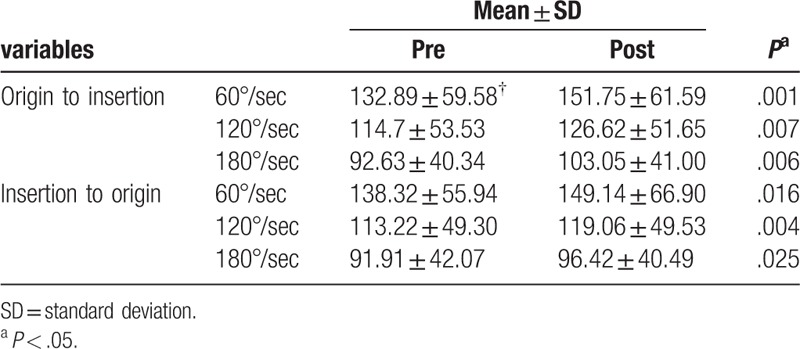
Change variable after intervention of quadriceps muscle peak torque.

### 3.3 Comparison of differences in peak torque according to kinesiology tape application direction

A comparison of the amount of change in the peak torques at the angular velocities of 60°/s, 120°/s, and 180°/s did not significantly differ between kinesiology tape application direction conditions (from the origin to the insertion vs from the insertion to the origin) (Table 3). At 60°/s, the peak torque change from the origin to insertion was 18.86 ± 19.76 and that from the insertion to origin was 10.82 ± 17.17 (*P* = .201). At 120°/s, the peak torque change from the origin to insertion was 11.92 ± 16.37 and that from the insertion to origin was 5.84 ± 7.48 (*P* = .164). At 180°/s, the peak torque change from the origin to insertion was 10.42 ± 14.26 and that from the insertion to origin was 4.51 ± 7.78 (*P* = .132). There was no significant difference in the amount of change in the peak torques according to the direction of kinesiology tape application, at the angular velocities of 60°/s, 120°/s, and 180°/s.

**Table 3:**
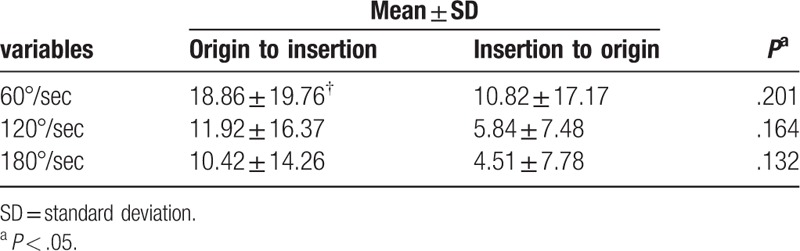
Comparison of the differences in quadriceps muscle peak torque between the taping direction conditions (from the origin to the insertion and from the insertion to the origin).

## 4 Discussion

Regardless of the application direction, there was a significant difference in the quadriceps peak torque before and after kinesiology tape application. In a study conducted in jockeys, Kim and Lee^[29]^ reported that muscle torque, as assessed by isokinetic equipment, increased after kinesiology tapes were applied to the quadriceps and hamstring. Similarly, Yeung et al^[30]^ observed a temporary increase in the isomorphic strength when kinesiology tape was applied to the vastus medialis oblique. The authors argued that the application of elastic kinesiology tape stimulated muscle spindles and facilitated muscle contraction via stretch mechanoreceptors. According to cutaneous fusimotor reflex theory, various tape of tactile stimulation, such as contact and vibration, induces gamma motor reflexes and increase muscle strength.^[31]^ Konishi^[2]^ argued that quadriceps strength improved because tactile stimulation due to kinesiology tape application affects gamma motor neurons by weakening Ia inhibitory afferent stimulation, and additionally facial contractibility functions to transport force. We also believe that in the present study, regardless of the application direction, kinesiology tape application increased muscle strength by weakening Ia afferent stimulation via tactile stimulation, aiding muscle contraction.^[2]^

Previous studies, including those by Fu et al,^[32]^ Lins et al,^[33]^ Vercelli et al,^[17]^ Wong et al,^[15]^ Poon et al,^[34]^ and Korman et al,^[35]^ did not find a significant difference in quadriceps strength (assessed by isokinetic equipment) with kinesiology tape application. However, in these studies, kinesiology tape was applied to only one of the 4 muscles comprising the quadriceps (specifically, the rectus femoris).^[14,17,32–35]^

In contrast, the present study followed the procedures of Han et al^[27]^ and applied kinesiology tape to 3 of 4 muscles of the quadriceps (specifically, the rectus femoris, vastus medialis, and vastus lateralis), and we observed a significant difference in muscle torque with kinesiology tape application.

In the present study, the quadriceps peak torque did not significantly differ between the two directions of kinesiology tap application. Vercelli et al^[17]^ applied facilitation, inhibition, and sham taping to the quadriceps muscles and found no significance differences in muscle torque assessed using isokinetic equipment, single-leg-hop performance, or the Global Rating of Change Scale (GRCS). In Cai et al,^[36]^ electromyography activity of the wrist extensor, grip strength, and self-perceived performance did not significantly differ among facilitation taping, inhibition taping, and tapeless conditions. Similarly, in Bravi et al,^[37]^ there was no significant difference in wrist muscle activity according to kinesiology tape application direction. Serrão et al^[18]^ also reported that quadriceps activation during squatting exercises did not differ depending on the kinesiology tape application direction. Lastly, Au et al^[38]^ evaluated facilitation, inhibition, and sham taping in subjects with lateral epicondylitis, and failed to find a significant difference in pain, grip strength, or wrist extensor muscle activity. Thus, the present results regarding kinesiology tape application direction are consistent with those in numerous previous studies.

The present study has several limitations. First, the age of subjects was limited to the mid-20 seconds; thus, it is difficult to generalize the study findings to the entire age spectrum. Second, only the immediate effect of kinesiology tape application was assessed, and a long-term effect was not evaluated. Thirdly, the study sample did not include those with weakened quadriceps muscles or muscle fatigue. Fourthly, after attaching the kinesiology tape, it was difficult to measure electromyography because the kinesiology tape overlapped with the electromyography electrodes. Due to this, only the quadriceps peak torque was measured; thus, the effect of kinesiology tape application direction in such individuals is unknown. Future research should improve on these limitations. In particular, additional research is needed in subject with quadriceps muscle fatigue or weak muscle strength.

## 5 Conclusion

The application of kinesiology tape application to the rectus femoris, vastus medialis, and vastus lateralis of the quadriceps increased the muscle torque, regardless of the tape application direction. Therefore, to enhance quadriceps strength, we recommend the application of kinesiology tape to 3 of the muscles of the quadriceps (specifically, the rectus femoris, vastus medialis and vastus lateralis), irrespective of the tape application direction.

## Author contributions

**Conceptualization:** Im-Rak Choi.

**Data curation:** Im-Rak Choi.

**Formal analysis:** Im-Rak Choi.

**Investigation:** Im-Rak Choi, Jung-Hoon Lee.

**Methodology:** Im-Rak Choi, Jung-Hoon Lee.

**Project administration:** Im-Rak Choi.

**Resources:** Im-Rak Choi.

**Software:** Im-Rak Choi.

**Supervision:** Jung-Hoon Lee.

**Writing – original draft:** Im-Rak Choi, Jung-Hoon Lee.

**Writing – review & editing:** Im-Rak Choi, Jung-Hoon Lee.
